# The impact of long-term exercise intervention on heart rate variability indices: a systematic meta-analysis

**DOI:** 10.3389/fcvm.2025.1364905

**Published:** 2025-06-12

**Authors:** Wenxin Zhang, Siyuan Bi, Lin Luo

**Affiliations:** School of Physical Education, Guizhou Normal University, Guiyang, China

**Keywords:** heart rate variability (HRV), long-term exercise interventions, cardiovascular health, randomized controlled trials, exercise modalities

## Abstract

**Background:**

Heart rate variability (HRV) is a critical indicator for assessing autonomic nervous system (ANS) function and is closely associated with both cardiovascular and psychological health. Although previous studies have demonstrated that exercise interventions can improve HRV, their effects vary considerably depending on exercise type, intervention characteristics, and individual differences. This meta-analysis aims to evaluate the impact of long-term exercise interventions on HRV and to explore potential factors that influence these effects.

**Methods:**

A systematic search was conducted in PubMed, Cochrane Library, EBSCO, CINAHL, Web of Science, and Embase up to November 20, 2024, to identify randomized controlled trials (RCTs) examining the effects of long-term exercise interventions on HRV. Studies involving short-term interventions, non-original research, or incomplete data were excluded. Two researchers independently performed data extraction and quality assessment. In total, 34 studies involving 1,434 participants were included.

**Results:**

Long-term exercise interventions significantly reduced the LF/HF ratio (*P* < 0.05). Subgroup analyses indicated that the intervention effects were more pronounced in populations with existing health conditions and in interventions lasting ≥8 weeks. Aerobic training and resistance training demonstrated significant benefits.

**Conclusion:**

Long-term exercise interventions significantly reduce the LF/HF ratio and improve ANS balance. These effects, however, are influenced by individual health status, intervention design, and control group conditions. The high heterogeneity among the included studies and limited data on certain intervention characteristics warrant cautious interpretation of the findings. Future research should focus on conducting more high-quality RCTs to validate these results.

**Systematic Review Registration:**

https://www.crd.york.ac.uk/PROSPERO/myprospero, PROSPERO CRD42024541380.

## 1 Introduction

Heart Rate Variability (HRV) reflects the functional state of the autonomic nervous system (ANS), particularly the dynamic balance between sympathetic and parasympathetic activity (1). It should be emphasized that HRV does not represent the heart's intrinsic ability to regulate its own rate but rather reflects the regulation of cardiac activity by the ANS under various physiological and pathological conditions. Owing to its non-invasive nature, ease of measurement, and the extensive information it can provide, HRV has been widely applied in fields such as affective science, psychology, and exercise science to evaluate autonomic regulation, cardiovascular health, and the effects of exercise interventions (2, 3).

Studies have demonstrated that reduced HRV is closely associated with an increased risk of various cardiovascular diseases, including hypertension, heart failure, and coronary artery disease (4). Moreover, HRV can be employed to predict risks related to diabetes, autonomic dysfunction, and mental health disorders (5, 6). In the field of exercise science, HRV is regarded as a critical tool for monitoring exercise intensity, evaluating recovery levels, and optimizing training programs (7).

Despite its significant research and application value, existing studies on HRV exhibit notable methodological and outcome-related inconsistencies. Substantial differences exist in the analytical methods used to evaluate HRV, including time-domain analysis, frequency-domain analysis, and nonlinear analysis, each varying in sensitivity and interpretative capacity (7, 8). For example, time-domain indices, such as the standard deviation of NN intervals (SDNN) and the root mean square of successive differences (RMSSD), primarily reflect overall HRV levels and short-term parasympathetic activity. In contrast, frequency-domain indices, such as the low-frequency to high-frequency (LF/HF) ratio, focus on the relative balance between sympathetic and parasympathetic activity (9). However, the selection and interpretation of these indices often vary across studies, thereby limiting the comparability of results (6).

Additionally, factors such as respiratory rate, depth, and rhythm significantly influence HRV measurements (10). The complex coupling between respiration and heart rate means that changes in respiratory patterns can affect HRV, particularly during social, emotional, and cognitive tasks (11). For instance, studies have shown that social-emotional tasks can reduce respiratory variability, even in situations that elicit positive emotions, which may indirectly affect HRV assessments (12).

The effects of different types of exercise interventions on HRV also vary significantly. Aerobic exercise is generally associated with notable improvements in HRV, reflecting enhanced parasympathetic activity (13). In contrast, the effects of resistance training are more complex and may depend on factors such as training intensity and the individual's baseline health status (14). Other forms of exercise, such as interval training, require further investigation to clarify their impact on HRV. Furthermore, individual differences (e.g., age, gender, health status) play a significant role in modulating the effects of exercise interventions on HRV, with distinct patterns of HRV changes observed between healthy individuals and patients with cardiovascular diseases (15).

Recent studies have explored the cumulative effects of exercise interventions on heart rate variability (HRV) through evidence-based methods. Yang et al. conducted a network meta-analysis (NMA) and found that high-intensity interval training (HIIT) had the most significant improvement on SDNN and RMSSD, while combined training (CBT) and resistance training (RT) showed the best effects on LF and HF power, respectively (16). Amekran et al. further demonstrated through a systematic review and meta-analysis that exercise training significantly improved SDNN, RMSSD, and HF power in healthy adults, confirming the positive regulatory role of exercise on the autonomic nervous system (17). Disessa et al. found that exercise interventions significantly improved HRV indicators, particularly SDNN and RMSSD, in hemodialysis patients (15). However, these studies primarily focused on healthy populations or specific disease groups, lacking systematic analysis of the long-term effects of exercise interventions, especially across different ages, genders, and special populations. Additionally, existing research has been insufficient in comparing mind-body exercises (e.g., yoga) with other forms of exercise.

Building on previous research, this study aims to further elucidate the sustained effects of long-term exercise interventions on HRV through a systematic meta-analysis. The research will focus on the specific impacts of different exercise modalities (e.g., aerobic training, resistance training, high-intensity interval training, and mind-body exercises) on HRV, with a particular emphasis on multiple outcome indicators, including SDNN, RMSSD, LF, HF, and the LF/HF ratio. Additionally, the study will incorporate long-term exercise intervention research targeting both healthy individuals and patients to evaluate the effects of exercise interventions in these populations. Moreover, this study will explore the influence of factors such as age heterogeneity and BMI on the research outcomes, providing a more comprehensive evidence base for exercise-induced improvements in cardiac autonomic function and the reduction of cardiovascular risk in adults.

## 2 Methods

### 2.1 Protocol and registration

This study conducted a computer-assisted systematic literature search (Register number: CRD42024541380) in accordance with the Preferred Reporting Items for Systematic Reviews and Meta-Analyses (PRISMA) 2020 guidelines.

### 2.2 Study design

This study adopted a systematic meta-analytic approach to comprehensively evaluate the overall impact of long-term exercise interventions on Heart Rate Variability (HRV) indices. The analysis focused on the effects of various exercise modalities, including high-intensity interval training (HIIT), aerobic training (AT), resistance training (RT), and mind-body training (MBT), on multiple HRV parameters, such as SDNN (Standard Deviation of NN intervals), RMSSD (Root Mean Square of Successive RR Interval Differences), LF (Low-Frequency component), HF (High-Frequency component), and the LF/HF ratio. Table 1 provides a summary of the physiological significance of each HRV index, thereby offering insights into their roles in reflecting autonomic nervous system function. By examining changes in these indices, the study thoroughly assessed the influence of exercise interventions on autonomic regulation and explored potential variations across different exercise modalities and health populations.

**Table 1:** Intrinsic physiological significance of heart rate variability (HRV) indices.

HRV index	Intrinsic physiological significance
SDNN	Reflects the overall level of heart rate variability and represents the overall regulatory capacity of the autonomic nervous system on the heart. Higher SDNN values indicate better overall autonomic nervous function.
RMSSD	Reflects the root mean square of successive differences between adjacent RR intervals, primarily representing parasympathetic nervous activity. Higher RMSSD values suggest stronger parasympathetic nervous activity.
LF	Reflects the low-frequency component of heart rate variability, representing the combined regulatory effects of both sympathetic and parasympathetic nervous systems, but mainly associated with sympathetic nervous activity. Higher LF values indicate stronger sympathetic nervous activity.
HF	Reflects the high-frequency component of heart rate variability, primarily representing parasympathetic nervous activity and closely related to respiratory rate. Higher HF values suggest stronger parasympathetic nervous activity.
LF/HF ratio	Reflects the balance between sympathetic and parasympathetic nervous systems, representing the relative proportion of sympathetic and parasympathetic nervous activities. A higher LF/HF ratio indicates a relative predominance of sympathetic nervous activity, while a lower ratio suggests a relative predominance of parasympathetic nervous activity.

### 2.3 Data sources and search strategy

As of November 20, 2024, a comprehensive and systematic literature search was conducted for this study, utilizing major academic databases including PubMed, The Cochrane Library, EBSCO, CINAHL, Web of Science, and Embase. The objective was to evaluate the impact of various exercise interventions on HRV. The assessment process included a meticulous examination of titles, abstracts, and full texts to ascertain the relevance and eligibility of the identified literature for inclusion in this review. Detailed search methodologies, encompassing specific search terms, Boolean operators, and inclusion/exclusion criteria, are presented in Table 2. This approach facilitates a transparent, systematic, and reproducible literature selection process.

**Table 2:** Literature search strategy.

Retrieve content	Search query
HRV	((((((((ALL = (Heart rate variability)) OR ALL = (heart rate)) OR ALL = (Rate variability)) OR ALL = (heart rate variability)) OR ALL = (heart beat variability)) OR ALL = (heart rate variation)) OR ALL = (heart beat variation)) OR ALL = (autonomic nervous system)) OR ALL = (autonomic nervous system)
HIIT	(((((((((((((((ALL = (High-intensity interval training)) OR ALL = (High intensity interval training)) OR ALL = (Sprint interval training)) OR ALL = (HIIT)) OR ALL = (sit)) OR ALL = (Sprint interval training)) OR ALL = (Repeated sprint training)) OR ALL = (RST)) OR ALL = (Anaerobic interval training)) OR ALL = (Aerobic interval training)) OR ALL = (Tabata training)) OR ALL = (Maximal effort)) OR ALL = (High intensity)) OR ALL = (Exercise)) OR ALL = (High intensity exercise)) OR ALL = (Maximal effort exercise)
AT	((((ALL = (Aerobic training)) OR ALL = (endurance training)) OR ALL = (Cardiopulmonary training)) OR ALL = (Oxygen training)) OR ALL = (Aerobic endurance training)
RT	(((((Resistance training) OR ALL = (Resistance exercise)) OR ALL = (Resistance)) OR ALL = (Resistance exercise)) OR ALL = (Strength exercise)) OR ALL = (Strength training)
MBT	(((((ALL = (Mind-Body Training)) OR ALL = (Physical and mental exercises)) OR ALL = (tai chi)) OR ALL = (Tai chi chuan)) OR ALL = (a kind of traditional Chinese shadowboxing)) OR ALL = (yoga)

HIIT, high-intensity interval training; AT, aerobic training; RT, resistance training; MBT, mind-body training.

### 2.4 Inclusion and exclusion criteria

#### 2.4.1 Inclusion criteria

##### 2.4.1.1 Population

Studies that evaluated the effects of long-term exercise interventions on HRV in humans, with no restrictions on age, gender, or health status, were included.

##### 2.4.1.2 Interventions

Studies had to evaluate the effects of one of the following training modalities: HIIT, AT, RT, or MBT. The intervention was required to involve a frequency of at least once per week and a minimum duration of two weeks to assess its impact on autonomic nervous function.

##### 2.4.1.3 Control group

The control group consisted of participants who either maintained their usual lifestyle or treatment, or took part in other interventions not primarily targeting cardiovascular or autonomic adaptation.

##### 2.4.1.4 Outcomes

Studies needed to report at least one static HRV measurement lasting a minimum of 5 min. They also had to include at least one HRV domain—time-domain indices (e.g., SDNN, RMSSD) or frequency-domain indices (e.g., LF, HF, LF/HF)—to assess changes in autonomic nervous function.

##### 2.4.1.5 Study design

Only RCTs were included to ensure the quality and reliability of the results.

#### 2.4.2 Exclusion criteria

##### 2.4.2.1 Population restrictions

Studies involving participants with severe mobility impairments were excluded, as their condition could affect their ability to participate in certain training modalities. Additionally, the raw data of individual participants were not directly examined in this analysis, necessitating this exclusion.

Interventions: To more accurately evaluate the effects of single training modalities, studies combining multiple training methods were excluded.

##### 2.4.2.2 Language restrictions

Non-English language articles were excluded to ensure language homogeneity and consistency in research analysis.

##### 2.4.2.3 Study design

Retrospective studies and other non-randomized controlled trial designs were excluded.

##### 2.4.2.4 Acute interventions and data integrity

Studies focusing on acute interventions (i.e., short-term effects of a single training session) were excluded. Additionally, studies with incomplete outcome indicator information or those where the required data could not be obtained through author consultation were excluded.

### 2.5 Data extraction and processing

In accordance with meta-analysis guidelines, this study implemented a double-blind literature screening process, independently conducted by two researchers. Initially, studies were preliminarily screened based on titles and abstracts to identify those meeting the inclusion criteria or with unclear eligibility. Subsequently, full-text reviews were performed, during which each researcher independently assessed the eligibility of articles for inclusion. At the conclusion of each screening phase, discrepancies were resolved through discussion and consensus. Data were independently extracted from each included study using a standardized extraction form specifically designed for this review. Extracted information encompassed study characteristics (e.g., source, first author, publication year), participant details (e.g., age, gender, sample size), intervention specifics (e.g., type, frequency, intensity, duration, and control group conditions), and outcome measurement tools. Key outcomes, including mean values and standard deviations (SD) for time-domain (e.g., SDNN, RMSSD) and frequency-domain metrics (e.g., LF, HF, LF/HF), were also extracted. For studies with partially reported or graphically presented data, authors were contacted via email to obtain complete original datasets.

To ensure data extraction accuracy and consistency, discrepancies were addressed as follows: First, a standardized extraction form was developed, clearly defining variables to be extracted, and data were independently extracted by two researchers. Following extraction, results were compared, discrepancies were identified, and consensus was reached through discussion. In cases of unresolved disagreements, a third researcher arbitrated. For studies with missing or incomplete data, authors were contacted via email to obtain data information. Additionally, researchers underwent pre-extraction training and calibration exercises to ensure a uniform understanding of extraction protocols. Extracted data were subjected to multiple rounds of verification and cross-validation to ensure reliability. All discrepancies and their resolution methods were meticulously documented. To address potential inconsistencies in data formats and units across studies, the following measures were implemented: Variables were converted to standardized units (e.g., SDNN and RMSSD were converted to milliseconds). For studies with inconsistent or missing data formats, authors were contacted to obtain original datasets, followed by cross-validation. All converted data underwent rigorous verification to ensure accuracy and comparability.

### 2.6 Quality assessment

Independent researchers were responsible for data extraction and quality assessment. Included studies had to measure time-domain (SDNN, RMSSD) and frequency-domain (LF, HF, LF/HF) indices before and after the intervention. These indices served to assess changes in HRV. Efforts were made to contact authors via email to obtain raw data for studies that reported incomplete data or provided data solely in chart form. Studies that failed to provide these measurements were excluded to ensure the interpretability and significance of the meta-analysis. The assessment focused on the completeness and consistency of data, as well as the rigor of study design. The quality assessment results for each study were used in subsequent data analyses to ensure the accuracy and reliability of the findings.I.

### 2.7 Data analysis

All statistical analyses were performed using Review Manager (version 5.4, The Nordic Cochrane Centre) and Stata (version 16, StataCorp LLC). The meta-analysis separately measured common time-domain HRV indices (SDNN, RMSSD) and frequency-domain indices (LFnu, HFnu, LF/HF). The primary outcome measures were the mean (M) and standard deviation (SD) of these indices. For each study's intervention and control groups, post-intervention M ± SD and the number of participants were entered into Review Manager 5.4. These data were used to calculate the standardized mean difference between changes in the intervention and control groups for each study. For studies with inconsistent units, conversions were made to milliseconds (ms) for SDNN and RMSSD, and to natural logarithm (Ln) form for LFnu and HFnu, ensuring consistency in the analysis. A fixed-effect model was adopted when *p* < 0.1 and *I*^2^ < 50%; a random-effects model was employed when *p* ≥ 0.1 and *I*^2^ ≥ 50%, accompanied by subgroup analyses to explore sources of heterogeneity. For continuous data, depending on the consistency of measurement tools, either the weighted mean difference (WMD) or standardized mean difference (SMD) was used as the effect size. All effect sizes (ESs) were presented with 95% confidence intervals (95% CI) and interpreted based on the magnitude of the ESs. The level of significance was set at *p* < 0.05. If more than 10 studies were included, funnel plots were used to assess publication bias, and sensitivity analyses for time-domain and frequency-domain indices were performed using Stata 16 to evaluate the stability of the results.

## 3 Results

### 3.1 Study selection

**Figure 1:**
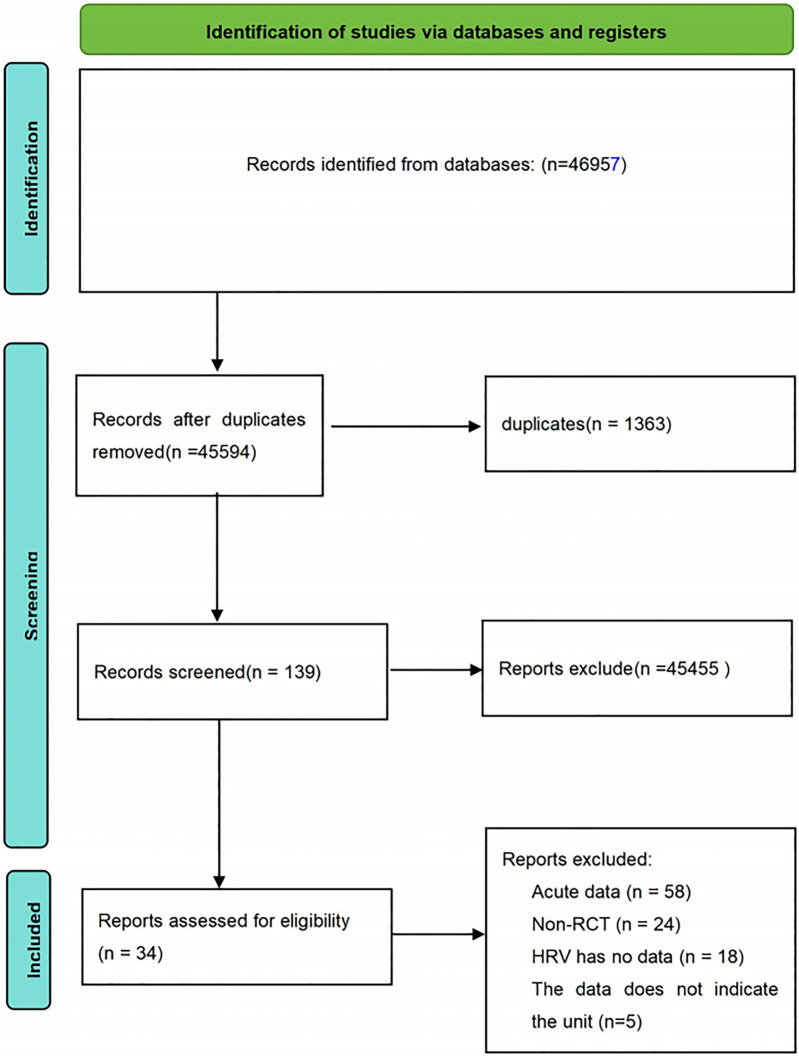
Procedure for study selection.

A comprehensive search of the main electronic databases yielded a total of 46,957 research records. After the removal of 1,363 duplicates, the remaining 45,594 articles underwent an initial screening process. This stage primarily involved filtering based on titles and abstracts, leading to the exclusion of 45,455 articles unrelated to the research theme. Consequently, 139 articles were deemed suitable for full-text evaluation. During the full-text review phase, 105 articles were excluded for failing to meet the predetermined inclusion criteria. These excluded articles consisted of studies with acute intervention data (58 articles), non-RCTs (24 articles), studies with incomplete HRV data (18 articles), and studies with unspecified outcome units (5 articles). Ultimately, 34 studies satisfied the established inclusion criteria and were selected for inclusion in this review. The detailed process of the literature search and screening is illustrated in Figure 1.

### 3.2 Characteristics of included studies

#### 3.2.1 Study design

This meta-analysis encompasses 34 studies, of which 25 utilized a randomized controlled trial design (7, 15–44), and 5 were multi-arm randomized parallel group controlled interventions (41–43, 45, 46), and 2 were single-blind randomized controlled trials (44, 47). Detailed study designs and control group conditions are presented in Table 3.

**Table 3:** Intervention characteristics.

Author (Year)	*n* (E: exercise group; C: control group	(Female/Male)	Participant age (Mean ± SD)	Research design	Intervention content	Frequency, periodicity	Participants or Subjects	Control Group Intervention Content	Outcome measures
A. Borghi-Silv et al. (2015)	E: 10	3/7	67 ± 7	RCT	AT	Three times/week, 12 weeks	Patients with chronic obstructive pulmonary disease (COPD)	No Exercise Interventions	Heart rate, oxygen consumption, carbon dioxide output, SDNN,RMSSD,LF,HF,LF/HF, etc.
	C: 10	5/5	66 ± 10						
A. M. Gerage et al. (2012)	E: 15	15/0	65.5 ± 5.0	RCT	RT	Three times/week, 12 weeks	Healthy elderly women	low-intensity activities	Resting systolic pressure, diastolic pressure, mean blood pressure, SDNN, RMSSD,LF,HF,LF/HF, etc.
	C: 14	14/0	66.2 ± 4.1						
Alireza Ghardashi-Afousi et al. (2018)	E: 14	0/14	53.90 ± 3.44	RCT	HIIT	Three times/week, 6 weeks	Men aged 50–70 who have undergone coronary artery bypass graft surgery in the past six weeks.	No Exercise Interventions	Maximum heart rate, resting heart rate, resting diastolic pressure, resting systolic pressure, RMSSD,LF/HF, etc.
	C: 14	0/14	58.80 ± 4.41						
Anneke van Biljon et al. (2018)	E: 29	42/67	11.07 ± 0.81	RCT	HIIT	Three times/week, 5 weeks	Children between the ages of 10 and 13 with no clinical evidence of known cardiovascular disease or any medical conditions that would limit exercise training.	No Exercise Interventions	SDNN,RMSSD,LF,HF,LF/HF
	C: 24								
Bandi Hari Krishna et al. (2014)	E: 44	12/32	49.34 ± 5.70	RCT	MBT	Three times/week, 12 weeks	Patients with heart failure	No Exercise Interventions	Heart rate, blood pressure, cardiac autonomic function LF,HF,LF/HF, and myocardial oxygen consumption, etc.
	C: 48	16/32	50.14 ± 4.54						
Birinder S. Cheema et al. (2013)	E: 18	14/4	37 ± 12	RCT	MBT	Three times/week, 10 weeks	Full-time scholars, staff, or graduate students	No Exercise Interventions	SDNN,RMSSD,LF/HF, musculoskeletal health (such as push-ups, side bridge, sit-and-reach tests), and psychological indicators (such as state and trait anxiety, quality of life, and job satisfaction), etc.
	C: 19	16/3	39 ± 13						
Bruna Gallo-Silva et al. (2019)	E: 10	Not specified	66.3 ± 6.5	RCT	AT	Three times/week, 8 weeks	Patients with chronic obstructive pulmonary disease (COPD)	No Exercise Interventions	SDNN,RMSSD,LF,HF,LF/HF
	C: 9		66.5 ± 9.5						
Djordje G. Jakovlevic et al. (2013)	E: 9	3/5	49 ± 13	RCT	RT	Three times/week, 8 weeks	Patients with non-alcoholic fatty liver disease (NAFLD)	No Exercise Interventions	LF,HF,LF/HF and autonomic and cardiac hemodynamic measurements at rest and submaximal exercise (50% of peak oxygen consumption), etc.
	C: 8	2/7	62 ± 7						
Gianluca Vernillo et al. (2015)	E: 9	9/0	24.3 ± 3.7	RCT	HIIT	Three times/week, 8 weeks	Male adults	No Exercise Interventions	SDNN,RMSSD
	C: 9	9/0							
Hélcio Kanegusuku, PhD et al. (2017)	E: 15	2/13	67 ± 8	Randomized parallel controlled trial	RT	Three times/week, 12 weeks	Patients with Parkinson's disease	No Exercise Interventions	Cardiac autonomic regulation (i.e., assessment of sympathetic and parasympathetic nervous system regulation of the LF,HF,LF/HF spectral analysis), etc.
	C: 16	5/11	68 ± 10						
I-Hua Chu et al. (2015)	E: 26	26/0	27.58 ± 6.12	RCT	MBT	Two times/week, 8 weeks	Healthy women	No Exercise Interventions	LF,HF,LF/HF with psychological measurement indicators.
	C: 26	26/0	24.85 ± 5.17						
I-Hua Chu et al. (2016)	E: 13	13/0	33.08 ± 9.11	RCT	MBT	Two times/week, 12 weeks	Women with depression	No Exercise Interventions	SDNN,LF,HF,LF/HF depression symptoms, and perceived stress.
	C: 13	13/0	32.38 ± 8.27						
Jesús Alarcón-Gómez et al. (2021)	E: 11	6/5	38 ± 5.5	Randomized parallel controlled trial	HIIT	Three times/week, 6 weeks	Type 1 diabetes patients	No Exercise Interventions	Maximum oxygen consumption, body composition, RMSSD,LF/HF, fasting blood sugar, etc.
	C: 8	4/4	35 ± 8.2						
Karavirta et al. (2013)	E: 26	26/0	52 ± 7	RCT	RT	Two times/week, 21 weeks	Middle-aged women	No Exercise Interventions	SDNN,LF/HF
	C: 17	17/0	52 ± 8						
Kellie Toohey et al. (2020)	E: 6	6/0	60 ± 8.12	RCT	HIIT	Three times/week, 12 weeks	Breast cancer survivors	No Exercise Interventions	Cardiopulmonary health (VO2 peak), resting RMSSD,LF,HF,LF/HF, and salivary biomarkers, etc.
	C: 6	6/0	61 ± 7.92						
Maharana Satyapriya et al. (2008)	E: 45	45/0	26.23 ± 2.98	Randomized two-arm experiment	MBT	Three times/week, 36 weeks	Healthy pregnant women	low-intensity activities	Perceived stress, cardiac autonomic response, LF,HF,LF/HF, etc.
	C: 45	45/0	25.47 ± 2.87						
Patricia Concepción García-Suárez et al. (2022)	E: 8	3/5	20.85 ± 1.7	RCT	HIIT	Three times/week, 8 weeks	Hypertensive patients	No Exercise Interventions	Muscle endurance and performance, SDNN,RMSSD,LF,HF,LF/HF, etc.
	C: 6	1/5	23.66 ± 2.6						
Preeyaphorn Songsorn et al. (2021)	E: 10	2/8	22.0 ± 0.8	RCT	HIIT	Three times/week, 6 weeks	Young individuals with low physical activity	No Exercise Interventions	HRV (time domain: SDNN and RMSSD, frequency domain: LF, HF, and LF/HF ratio), and resting heart rate, etc.
	C: 11	4/7	21.7 ± 0.8						
Ran L et al. (2022)	E: 13	13/0	22.6 ± 2.5	RCT	RT	Two times/week, 8 weeks	Non-professional, anxiety-prone female college students	No Exercise Interventions	BMI (Body Mass Index), self-assessment anxiety scale, SDNN,RMSSD,LF/HF, etc.
	C: 14	14/0	22.5 ± 2.0						
Salene M.W. Jones et al. (2017)	E: 100	100/0	54.4 ± 3.9	RCT	MBT	Ninety minutes/week,12 weeks	Women with symptoms of vascular constriction	No Exercise Interventions	SDNN,RMSSD,LF,HF
	C: 135	135/0	54.1 ± 3.5						
Shirley Telles et al. (2016)	E: 22	12/10	34.6 ± 6.5	Randomized two-arm experiment	MBT	One time/week, 3 months	Patients with chronic lower back pain	No Exercise Interventions	RMSSD,LF,HF,LF/HF
	C: 23	10/13	36.6 ± 6.0						
Shuai Zheng et al. (2017)	E: 17	11/6	35.4 ± 2.1	Randomized three-arm parallel controlled trial	MBT	Five hours/week,12 weeks	People who are healthy but have anxiety	No Exercise Interventions	State-Trait Anxiety Inventory, Perceived Stress Scale 14 (PSS14), blood pressure, and LF,HF, etc.
	C: 16	14/2	34.6 ± 2.3						
Sophie Cassidy et al. (2019)	E: 11	2/9	60 ± 3	RCT	HIIT	Three times/week,12 weeks	Type 2 diabetes patients	No Exercise Interventions	SDNN,LF,HF,LF/HF blood pressure variability, and sensitivity of pressure reflex receptors, etc.
	C: 11	3/8	59 ± 3						
Stefano Benítez-Flores et al. (2019)	E: 8	15/15	25.3 ± 5.3	RCT	HIIT	Three times/week,2 weeks	Healthy adults	No Exercise Interventions	Body composition, aerobic and anaerobic performance, SDNN,RMSSD, and redox status, etc.
	C: 6		26.2 ± 3.9						
Stella V. Philbois et al. (2022)	E: 25	25/0	29 ± 4	RCT	HIIT	Three times/week,16 weeks	Patients with polycystic ovary syndrome (PCOS)	No Exercise Interventions	Cardiopulmonary tests, RMSSD,LF,HF,LF/HF, blood pressure variability (BPV), and spontaneous baroreflex sensitivity (BRS), etc.
	C: 25	25/0	29 ± 5						
Stephen H. Boutcher et al. (2013)	E: 16	16/0	23.0 ± 4.0	RCT	HIIT	Three times/week,12 weeks	Non-smoking, non-exercising, overweight young women who are healthy	No Exercise Interventions	Peak oxygen consumption (VO_2_ peak), heart rate,LF,HF, etc.
	C: 16	16/0	21.1 ± 2.4						
Steve E. Selig, PhD et al. (2003)	E: 19	4/15	65 ± 13	RCT	RT	Three times/week,12 weeks	Patients with chronic heart failure (CHF)	No Exercise Interventions	VO_2_ peak, resting SDNN,RMSSD,LF,HF,LF/HF, blood pressure variability, etc.
	C: 20	2/18	64 ± 9						
Tseng-Hau Tseng et al. (2017)	E: 20	15/5	61.1 ± 6.8	RCT	AT	Three times/week,12 weeks	Adults aged ≥40 with poor sleep quality	No Exercise Interventions	Subjective (Pittsburgh Sleep Quality Index, PSQI) and objective (actigraphy recording) sleep quality assessment, SDNN,LF,HF,LF/HF etc.
	C: 20	18/2	62.2 ± 7.4						
Wan-An Lu et al. (2011)	E: 25	17/18	57 ± 4.25	RCT	MBT	40 min/week, 3 months	Patients with chronic heart failure	No Exercise Interventions	Lung function, glucose availability, blood lipid levels, and LF,HF, etc.
	C: 25	13/12	53 ± 3						
Wei Zhou et al. (2017)	E: 57	19/38	<30 years old (*n* = 20);	RCT	MBT	Five times/week, 7 weeks	Patients with nasopharyngeal carcinoma	No Exercise Interventions	Scoring on the Multidimensional Fatigue Symptom Inventory. Heart rate variability parameters, including normalized low-frequency (nLF) power, normalized high-frequency (nHF) power, and their correlations with CRF (Cardiorespiratory Fitness), etc.
	C: 57	12/45	30–50 years old (*n* = 71)						
Sascha Ketelhut（2024）	E: 20	10/10	11 ± 0.6	RCT	HIIT	Two times/week,12 weeks	Elementary school students	low-intensity activities	SDNN,RMSSD,LF,HF,LF/HF
	C: 20	7/13	11 ± 0.7						
Blake E. G. Collins（2023）	E: 15	0/15	51.1 ± 5.7	RCT	AT	Three times/week, 12 weeks	Sedentary men with type 2 diabetes	No Exercise Interventions	Cardiorespiratory fitness, SDNN,RMSSD,flow-mediated dilation
Ganagarajan Inbaraj（2023）	C: 15	0/15	51.2 ± 7	Single-blind randomized controlled trial	MBT	Five times/week,18 weeks	Patients with stage I, II, and III breast cancer		
	E: 29	29/0	45.4 ± 7.7						
	No Exercise Interventions	RMSSD,LF,HF,LF/HF							
	C: 30	30/0	46.3 ± 6.1						
Pooja Bhati（2023）	E: 28	15/13	52.8 ± 6.82	Single-blind randomized controlled trial	RT	Three times/week,12 weeks	Patients with type 2 diabetes	No Exercise Interventions	SDNN,RMSSD,LF,HF,LF/HF, heart rate recovery, spontaneous baroreflex sensitivity, biochemical analysis of biomarkers
	C: 28	17/11	54.0 ± 8.18						

HIIT, high-intensity interval training; AT, aerobic training; RT, resistance training; MBT, mind-body training.

#### 3.2.2 Participants

The included studies comprised a total of 1,434 participants, ranging in age from 11 ± 0.6 to 67 ± 7 years. Of these, 5 studies focused on the elderly population (aged ≥65 years) (15, 18, 42, 48, 49), 2 targeted children and adolescents (aged 10–17 years) (22, 39), and 19 aimed at adults (aged 18–65 years) (17, 19, 20, 22, 25, 26, 28, 30, 33, 34, 36, 41, 44–47, 50–52). In terms of gender, 9 were female-only (18, 20, 25, 30, 34, 44, 45, 52, 53), and 15 included both males and females (7, 15, 17, 19, 23, 26, 28, 33, 39, 41, 42, 47, 50, 51). 12 studies involved healthy populations (17, 18, 20, 21, 26, 33, 34, 39, 45, 50, 53), while 14 focused on patients with conditions like hypertension diabetes, various forms of heart failure, and chronic obstructive pulmonary disease, which are often associated with autonomic cardiac dysfunction (19, 22, 25, 28, 30, 41, 42, 44, 46–49, 51, 54).

#### 3.2.3 Interventions

The analyzed studies implemented at least one of the training modalities such as HIIT, AT, RT, or MBT. HIIT was the primary intervention in 8 studies (22, 23, 25, 26, 28, 39, 41, 50), encompassing regular circuit training, power cycling, treadmill exercises, etc.; 3 studies used AT (15, 17, 48), primarily in the form of brisk walking or jogging; 7 studies adopted RT (18–20, 42, 47, 49, 52); and 7 studies incorporated MBT (33, 34, 44–46, 51, 53), such as yoga or Tai Chi. The majority of studies had an intervention frequency of ≥3 times per week and a duration of ≥8 weeks.

#### 3.2.4 Outcome measures

All studies reported at least one objective HRV measurement. 19 studies involved SDNN (7, 15, 17, 18, 20, 21, 23, 24, 26, 28, 29, 33, 35, 39, 40, 47, 48, 50, 53), 21 addressed RMSSD (15, 18, 21–26, 29, 30, 33, 35, 39–41, 44, 46–50), twenty-three focused on LF and HF (15–19, 23, 25, 27, 28, 30–32, 34, 35, 38, 42, 43, 45, 46, 49, 53), and 23 included LF/HF ratio (15, 17–19, 23, 25, 26, 28, 30, 32, 34, 35, 38, 39, 42–44, 46–51, 53). The majority of the studies used Polar Pro straps or other Polar devices for measurement, a few employed Suunto devices, while others utilized computer and commercial systems (Oxford Instruments, Excel ECG Playback System-Rel8.5), portable ECG Holter monitors worn for 24 h (Tracker, Reynolds Medical Ltd., Hertfordshire, UK), ultrasonic monitors (HRV, 8Z11, Wegene Technology, Taiwan), Powerlab data acquisition systems (Data Acquisition Corporation, Colorado Springs, CO, USA), digital ECGs (Micromed, sampling rate: 250 Hz), (UFI, Pneumotrace 2, 1998, California, USA), among other instruments for sampling. One study did not specify the measurement tool used.

### 3.3 Risk of bias

**Figure 2:**
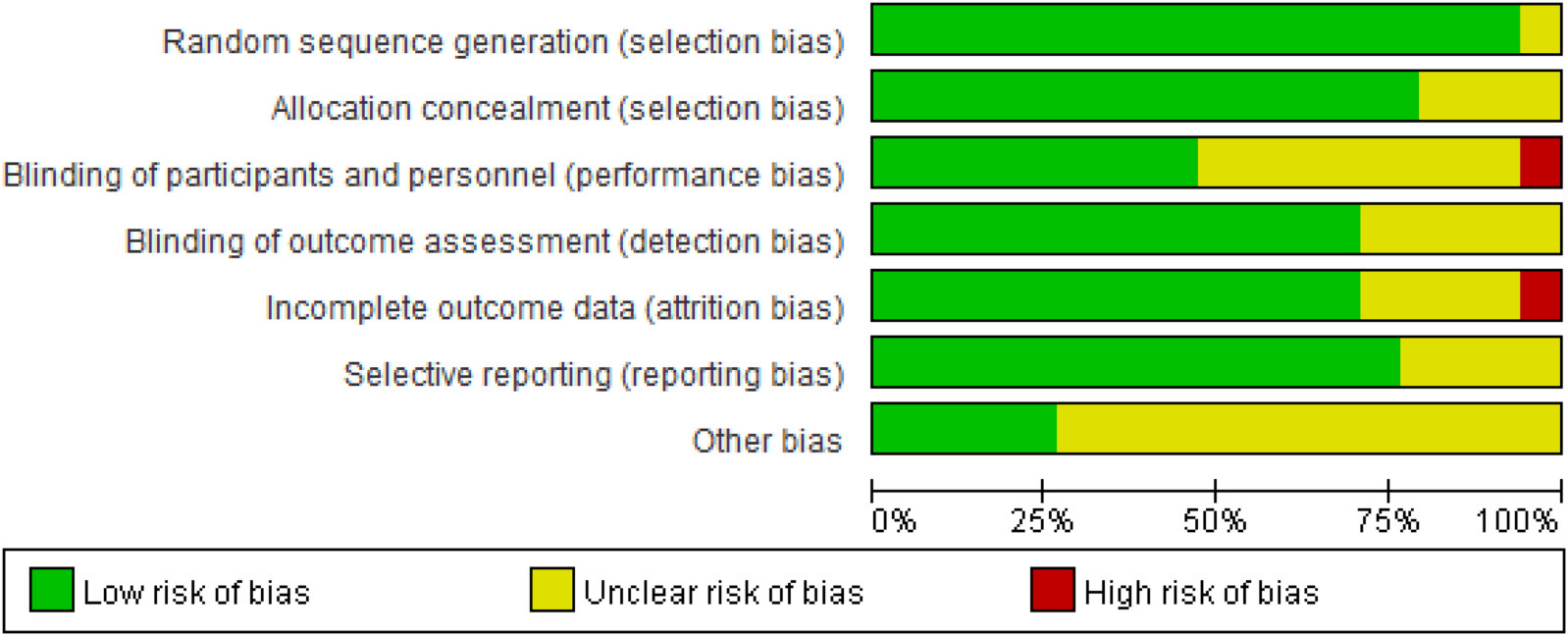
Proportion chart of literature quality assessment.

The quality of the included studies was assessed using the Cochrane Collaboration's tool in Review Manager 5.4. This process was independently conducted by two reviewers and covered seven domains (Figure 2): selection bias, performance bias, detection bias, attrition bias, reporting bias, and other potential sources of bias. The risk of bias in each domain was categorized as “low risk,” “high risk,” or “unclear risk.”

The criteria for “low risk” included the following: clear randomization methods and allocation concealment to ensure the grouping process was not compromised; the use of double-blind designs, where both investigators and participants were blinded to group assignments; clear blinding of outcome assessors to prevent subjective judgments from influencing results; complete follow-up data with low attrition rates (typically below 20%) or sensitivity analyses indicating that attrition had no significant impact; pre-registration of study protocols with all pre-specified outcomes reported, avoiding selective reporting; and no other sources of bias that could affect results, such as imbalances in baseline characteristics or undisclosed conflicts of interest. Domains meeting these criteria were rated as “low risk,” while those failing to meet the criteria were rated as “high risk” or “unclear risk.”

**Figure 3:**
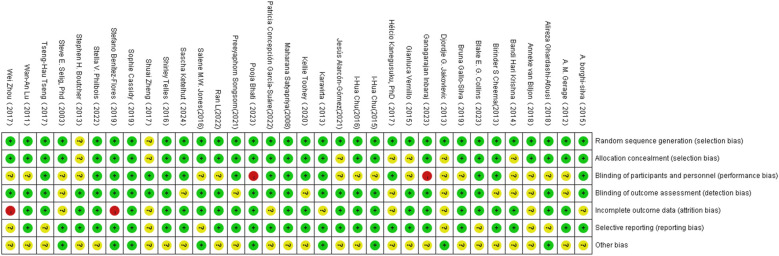
Illustration of literature quantity assessment. Note: A plus sign (+) indicates high methodological quality (low risk of bias); a minus sign (−) indicates low methodological quality (high risk of bias); a question mark (?) indicates unclear methodological quality (insufficient reporting on what occurred during the study).

The results of the risk of bias assessment are presented in Figure 3.

### 3.4 Main research findings

#### 3.4.1 Time-Domain analysis

**Figure 4:**
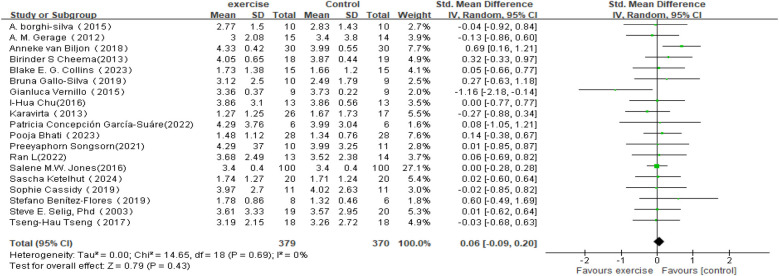
Forest plot of the meta-analysis on the effects of long-term exercise intervention on SDNN.

In the analysis of changes in SDNN (see Figure 4), a total of 19 studies involving 749 participants were included to compare post-intervention SDNN changes between the exercise intervention group and the control group. The meta-analysis indicated that the improvement in SDNN in the intervention group was minimal compared to the control group and did not reach statistical significance (SMD = 0.06, 95% CI: −0.09 to 0.20, *P* = 0.43). Moreover, the overlapping confidence intervals between groups suggested that the difference may not be significant. Heterogeneity analysis revealed low variability among the included studies (*I*^2^ = 0%).

**Figure 5:**
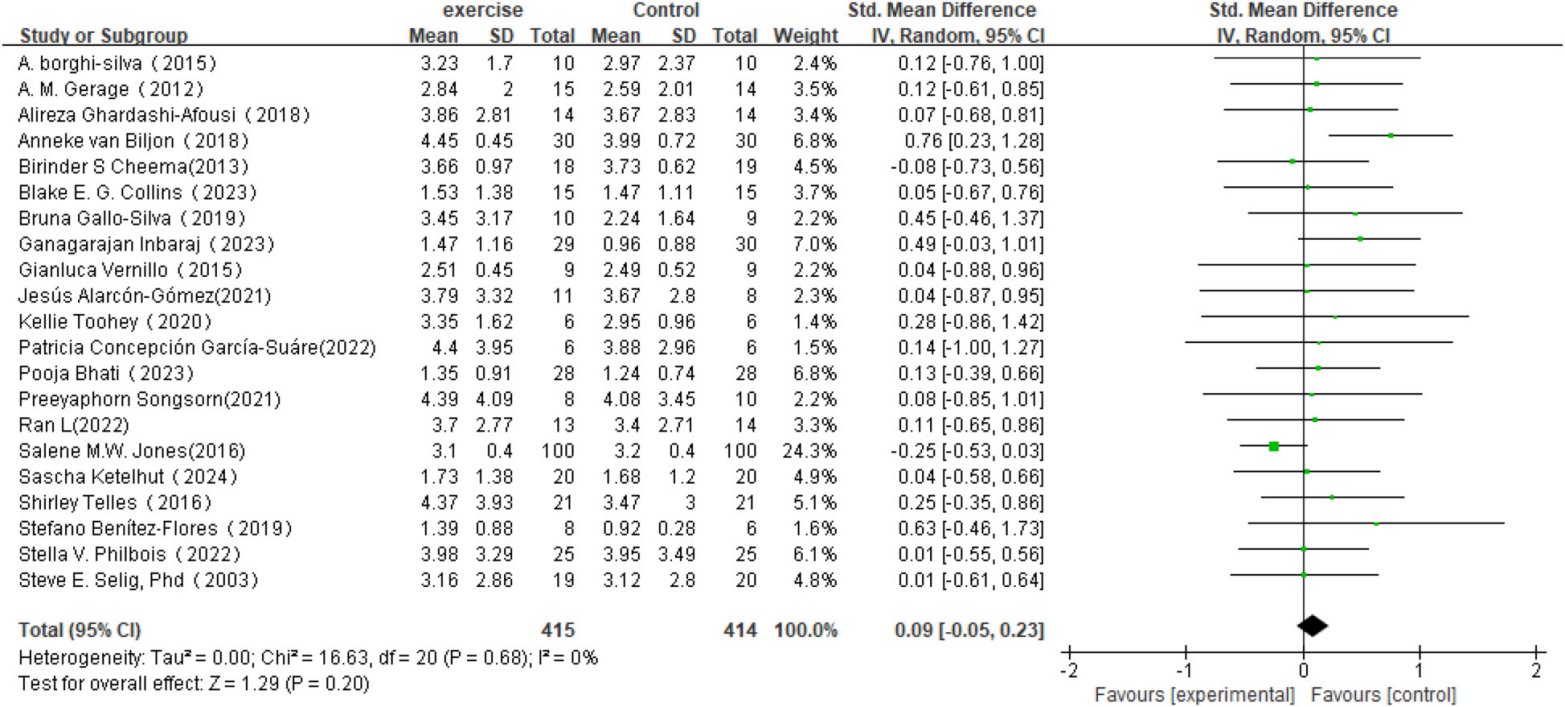
Forest plot of the meta-analysis on the effects of long-term exercise intervention on RMSSD.

Regarding changes in RMSSD in HRV (see Figure 5), this meta-analysis included 21 studies involving 829 participants. The results showed that the effect of exercise intervention on RMSSD was small compared to the control group (SMD = 0.09, 95% CI: −0.05 to 0.23), and it did not reach statistical significance (*P* = 0.20).Heterogeneity analysis indicated low variability among the included studies (*I*^2^ = 0%).

#### 3.4.2 Frequency-domain analysis

**Figure 6:**
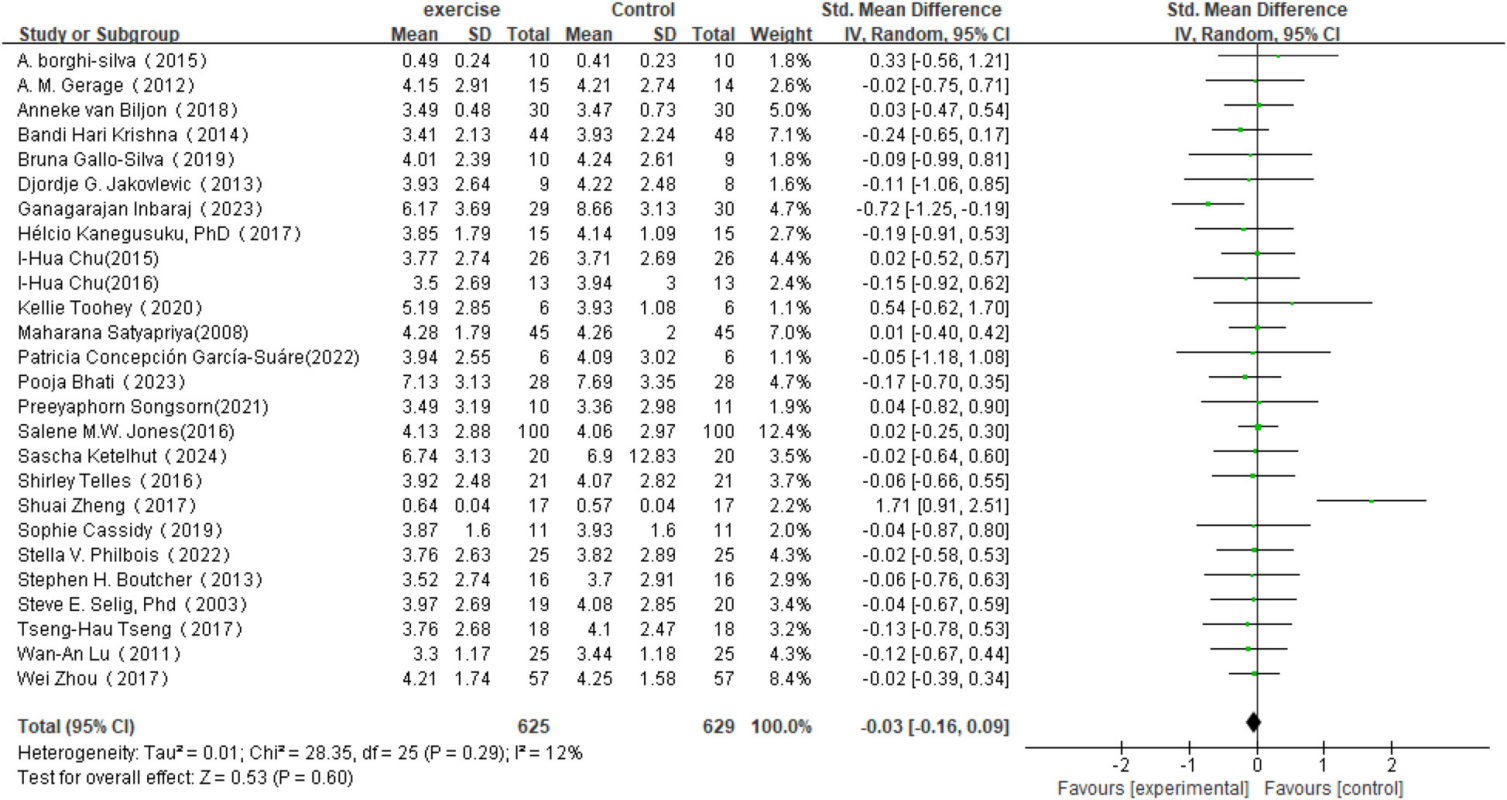
Forest plot of the meta-analysis on the effects of long-term exercise intervention on LF.

In the analysis of post-intervention LF changes (see Figure 6), a total of 26 studies involving 1,254 participants were included. The meta-analysis indicated a very small SMD between the intervention and control groups (SMD = −0.03, 95% CI: −0.16 to 0.09), which did not reach statistical significance (*P* = 0.60). Heterogeneity analysis revealed low variability among the included studies (*I*^2^ = 12%).

**Figure 7:**
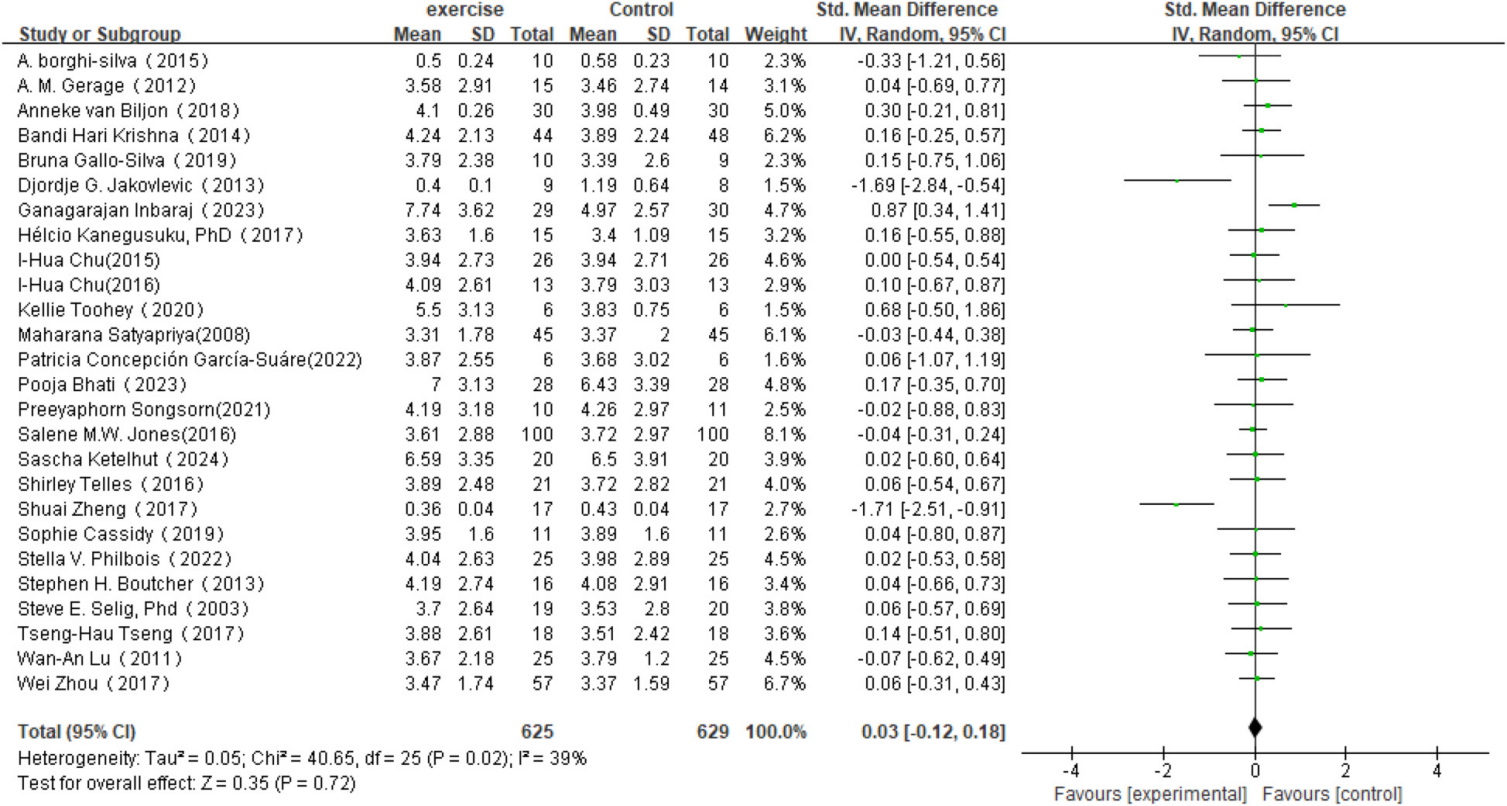
Forest plot of the meta-analysis on the effects of long-term exercise intervention on HF.

In the analysis of post-intervention HF changes (see Figure 7), a total of 26 studies involving 1,254 participants were included. The results showed an SMD of 0.03 (95% CI: −0.12 to 0.18) between the intervention and control groups, which did not reach statistical significance (*P* = 0.72). The heterogeneity among studies was 39%, indicating a moderate level of variability.

**Figure 8:**
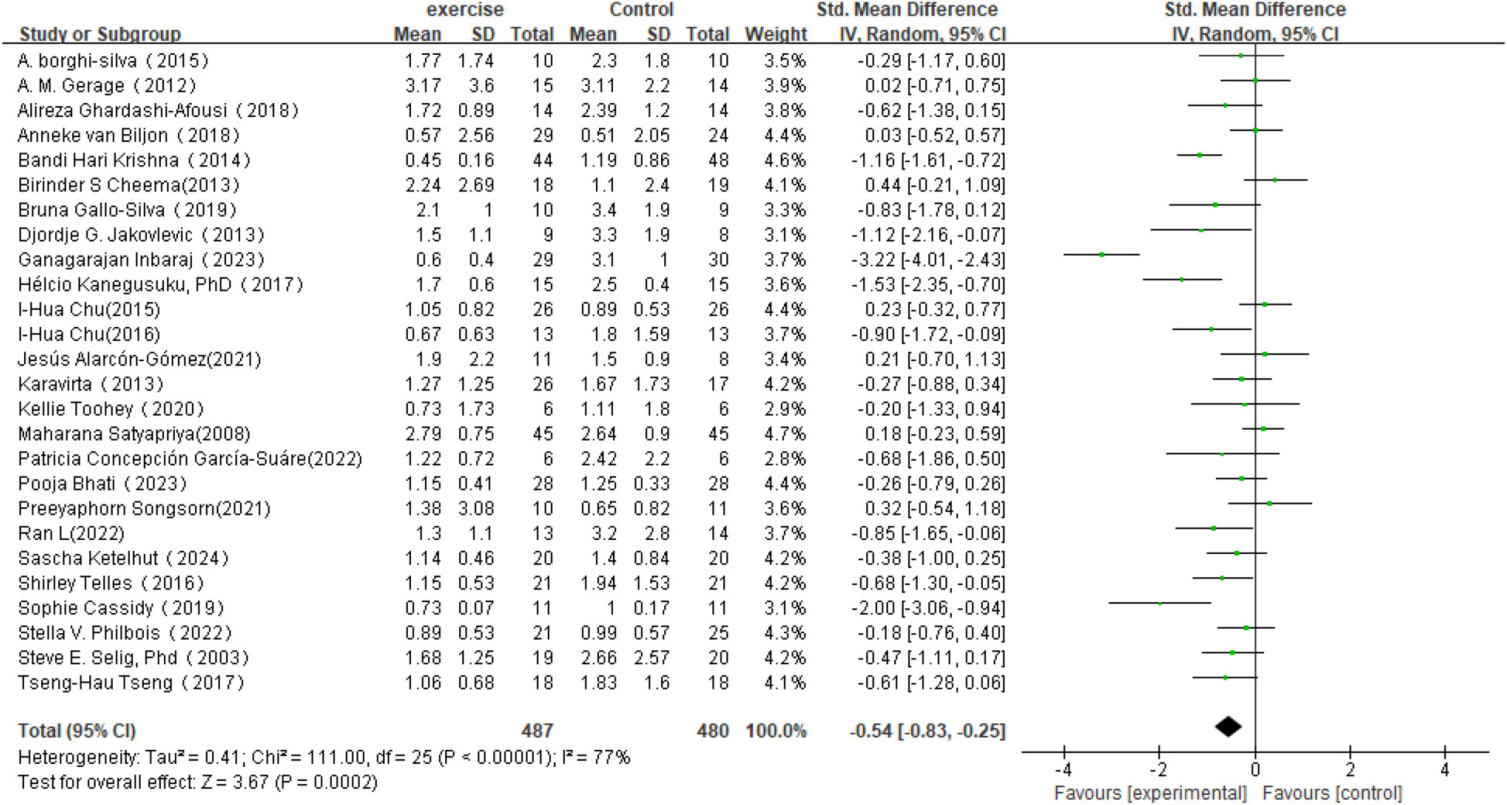
Forest plot of the meta-analysis on the effects of long-term exercise intervention on LF/HF ratio.

In the analysis of post-intervention LF/HF changes, a total of 26 studies involving 967 participants were included (see Figure 8). The meta-analysis showed that LF/HF in the intervention group was significantly lower than in the control group (SMD = −0.54, 95% CI: −0.83 to −0.25, *P* = 0.0002). A reduction in LF/HF indicates decreased sympathetic nervous system activity relative to parasympathetic nervous system activity, suggesting that exercise interventions may improve the balance of the autonomic nervous system. However, despite the significant mean difference observed in the intervention group, heterogeneity among studies was high (*I*^2^ = 77%). Therefore, further subgroup or sensitivity analyses are planned to identify sources of heterogeneity and to explore the effects of different intervention types, sample characteristics, or study designs on the results.

### 3.5 Subgroup analysis of LF/HF ratio

#### 3.5.1 Individual factors

Table 4 presents the effects of different subgroups on LF/HF and their heterogeneity.

**Table 4:** Subgroup analysis results.

Subgroup	Group	Std	Mean Difference (IV, Random, 95% CI)	*p*-value	Heterogeneity (*I*^2^%)	Between-group *p*-value	Between-group heterogeneity (*I*^2^%)
Individual factors							
Age group	24	−0.58	[−0.89,−0.26]			0.94	0%
18–65 years	19	−0.57	[−0.94,−0.20]	0.003	82%		
>65 years	5	−0.60	[−1.11,−0.08]	0.02	53%		
Health status	26	−0.54	[−0.83,−0.25]			0.004	87.8%
Healthy	12	−0.14	[−0.38,0.11]	0.27	39%		
Disease	14	−0.87	[−1.31,−0.43]	<0.001	80%		
Gender	24	−0.53	[−0.83,−0.22]			0.90	0%
Female only	9	−0.56	[−1.19,0.08]	0.09	88%		
Mixed gender	15	−0.60	[−1.11,−0.08]	0.002	67%		
BMI	20	−0.61	[−0.97,−0.26]			0.40	0%
Normal	6	−0.37	[−0.75,0.01]	0.06	46%		
Overweight	10	−0.63	[−1.26,−0.00]	0.05	88%		
Obese	4	−0.95	[−1.74,−0.15]	0.02	67%		
Region	25	−0.57	[−0.87,−0.27]			0.08	51.3%
South America	5	−0.52	[−1.07,0.02]	0.06	59%		
Asia	10	−0.62	[−1.18,−0.06]	0.03	87%		
North America	2	−1.10	[−1.52,0.69]	<0.001	0%		
Europe	5	−0.63	[−1.27,0.00]	0.05	67%		
Oceania	3	−0.06	[−0.68,0.56]	0.85	49%		
Study design							
Intervention content	25	−0.56	[−0.86,−0.26]			0.84	0%
HIIT	8	−0.36	[−0.80,0.09]	0.11	55%		
Aerobic training	3	−0.57	[−1.04,−0.11]	0.02	0%		
Resistance training	7	−0.56	[−0.93,−0.19]	0.003	47%		
Mind-body training	7	−0.71	[−1.50,0.09]	0.08	93%		
Intervention duration	26	−0.54	[0.83,−0.25]			0.02	81.9%
≥8 weeks	22	−0.63	[−0.96,−0.31]	<0.001	79%		
<8 weeks	4	−0.04	[−0.42,0.34]	0.84	7%		
Intervention frequency	26	−0.54	[−0.83,−0.25]			0.39	0%
≥3 times/week	19	−0.52	[−0.90,−0.15]	0.006	81%		
<3 times/week	7	−0.57	[−0.98,−0.16]	0.007	61%		
Control group	26	−0.54	[−0.83,−0.25]			0.008	86%
No exercise interventions	23	−0.61	[−0.93,−0.29]	<0.001	78%		
Low-intensity activities	3	0.01	[−0.32,0.33]	0.97	5%		

A total of 24 studies were included in the subgroup analysis of age effects on LF/HF. In the 18–65 age group, SMD = −0.57 (95% CI: −0.94 to −0.20, *P* = 0.003, *I*^2^ = 82%). In the 65+ age group, SMD = −0.60 (95% CI: −1.11 to −0.08, *P* = 0.02, *I*^2^ = 53%). Although the exercise intervention showed a significant effect on the LF/HF ratio in the 18–65 age group and the group over 65 years, the between-group comparison with a *p*-value of 0.94 indicates that the impact of the exercise intervention on the two age groups is not significantly different.

A total of 26 studies were included in the subgroup analysis of health status effects on LF/HF. In healthy individuals, SMD = −0.14 (95% CI: −0.38 to 0.11, *P* = 0.27, *I*^2^ = 39%). In individuals with illnesses, SMD = −0.87 (95% CI: −1.31 to −0.43, *P* < 0.001, *I*^2^ = 80%). The study results indicate that the exercise intervention effect in the healthy group is not significant compared to the control group, whereas the effect in the disease group is significant. The between-group comparison yielded a *p*-value of 0.004, and the confidence intervals for the two groups do not overlap, indicating a significant difference in the intervention outcomes between the healthy and disease groups.

A total of 24 studies were included in the subgroup analysis of gender effects on LF/HF. In the female group, SMD = −0.56 (95% CI: −1.19 to 0.08, *P* = 0.09, *I*^2^ = 88%). In the mixed-gender group, SMD = −0.60 (95% CI: −1.11 to −0.08, *P* = 0.002, *I*^2^ = 67%). The study results showed that the exercise intervention had a significant effect on the LF/HF ratio in the mixed-gender group, but the effect was not statistically significant in the female group.

A total of 20 studies were included in the subgroup analysis of BMI effects on LF/HF. In the normal group, SMD = −0.37 (95% CI: −0.75 to 0.01, *P* = 0.06, *I*^2^ = 46%). In the overweight group, SMD = −0.63 (95% CI: −1.26 to 0.00, *P* = 0.05, *I*^2^ = 88%). In the obese group, SMD = −0.95 (95% CI: −1.74 to −0.15, *P* = 0.02, *I*^2^ = 67%). The study results showed that the exercise intervention had a significant effect on the LF/HF ratio in the obesity group, but did not demonstrate significant effects in the normal weight and overweight groups.

A total of 22 studies were included in the subgroup analysis of regional effects on LF/HF. In South America, SMD = −0.52 (95% CI: −1.07 to 0.02, *P* = 0.06). In Asia, SMD = −0.62 (95% CI: −1.18 to −0.06, *P* = 0.03). In North America, SMD = −1.10 (95% CI: −1.52 to −0.69, *P* < 0.001). In Europe, SMD = −0.63 (95% CI: −1.27 to 0.00, *P* = 0.05). In Oceania, SMD = −0.06 (95% CI: −0.68 to 0.56, *P* = 0.85). The study results showed that the exercise intervention had statistically significant effects in the Asian and North American subgroups, but did not reach statistical significance in the South American, Oceania, and European subgroups.

#### 3.5.2 Intervention characteristics

A total of 25 studies were included in the subgroup analysis of training methods on LF/HF. For HIIT, SMD = −0.36 (95% CI: −0.80 to 0.09, *P* = 0.11, *I*^2^ = 55%). For aerobic training, SMD = −0.57 (95% CI: −1.04 to −0.11, *P* = 0.02, *I*^2^ = 0%). For resistance training, SMD = −0.56 (95% CI: −0.93 to −0.19, *P* = 0.003). For mind-body training, SMD = −0.71 (95% CI: −1.50 to 0.09, *P* = 0.08, *I*^2^ = 93%). The study results indicated that compared to the control group, High-Intensity Interval Training (HIIT) and mind-body interventions did not show significant effects. In contrast, aerobic training and resistance training demonstrated positive intervention outcomes.

A total of 26 studies were included in the subgroup analysis of intervention duration on LF/HF. For interventions ≥8 weeks, SMD = −0.63 (95% CI: −0.96 to −0.31, *P* < 0.001, *I*^2^ = 79%). For interventions <8 weeks, SMD = −0.04 (95% CI: −0.42 to 0.34, *P* = 0.84, *I*^2^ = 7%). The study results indicate that, compared to the control group, exercise interventions lasting ≥8 weeks significantly influenced the LF/HF ratio, whereas interventions lasting <8 weeks showed no significant effect. The between-group comparison yielded a *p*-value of 0.02; however, due to partial overlap in the confidence intervals, the results should be interpreted with caution.

A total of 26 studies were included in the subgroup analysis of intervention frequency on LF/HF. For interventions ≥3 times/week, SMD = −0.52 (95% CI: −0.90 to −0.15, *P* = 0.006, *I*^2^ = 81%). For interventions <3 times/week, SMD = −0.57 (95% CI: −0.98 to −0.16, *P* = 0.007, *I*^2^ = 61%). The study results indicate that, compared to the control group, exercise interventions with frequencies of ≥3 times per week and <3 times per week both significantly influenced the LF/HF ratio. The between-group comparison yielded a *p*-value of 0.39, suggesting that the differences in the effects of intervention frequency on the LF/HF ratio were not statistically significant.

A total of 26 studies were included for subgroup analysis against control groups. For the intervention group compared to the “No Exercise Interventions” control group, the standardized mean difference (SMD) was −0.61 (95% CI: −0.93 to −0.29, *P* < 0.001, *I*^2^ = 78%). For the intervention group compared to the “low-intensity activities” control group, the SMD was 0.01 (95% CI: −0.32 to 0.33, *P* = 0.97, *I*^2^ = 5%). The results indicate that the effect of exercise intervention on the LF/HF ratio was significantly more pronounced compared to the “No Exercise Interventions” control group. However, no significant effect was observed when compared to the “low-intensity activities” control group. Although the between-group comparison yielded a *p*-value of 0.008, the partial overlap in the confidence intervals suggests that the results should be interpreted with caution

### 3.6 Sensitivity analysis

**Figure 9:**
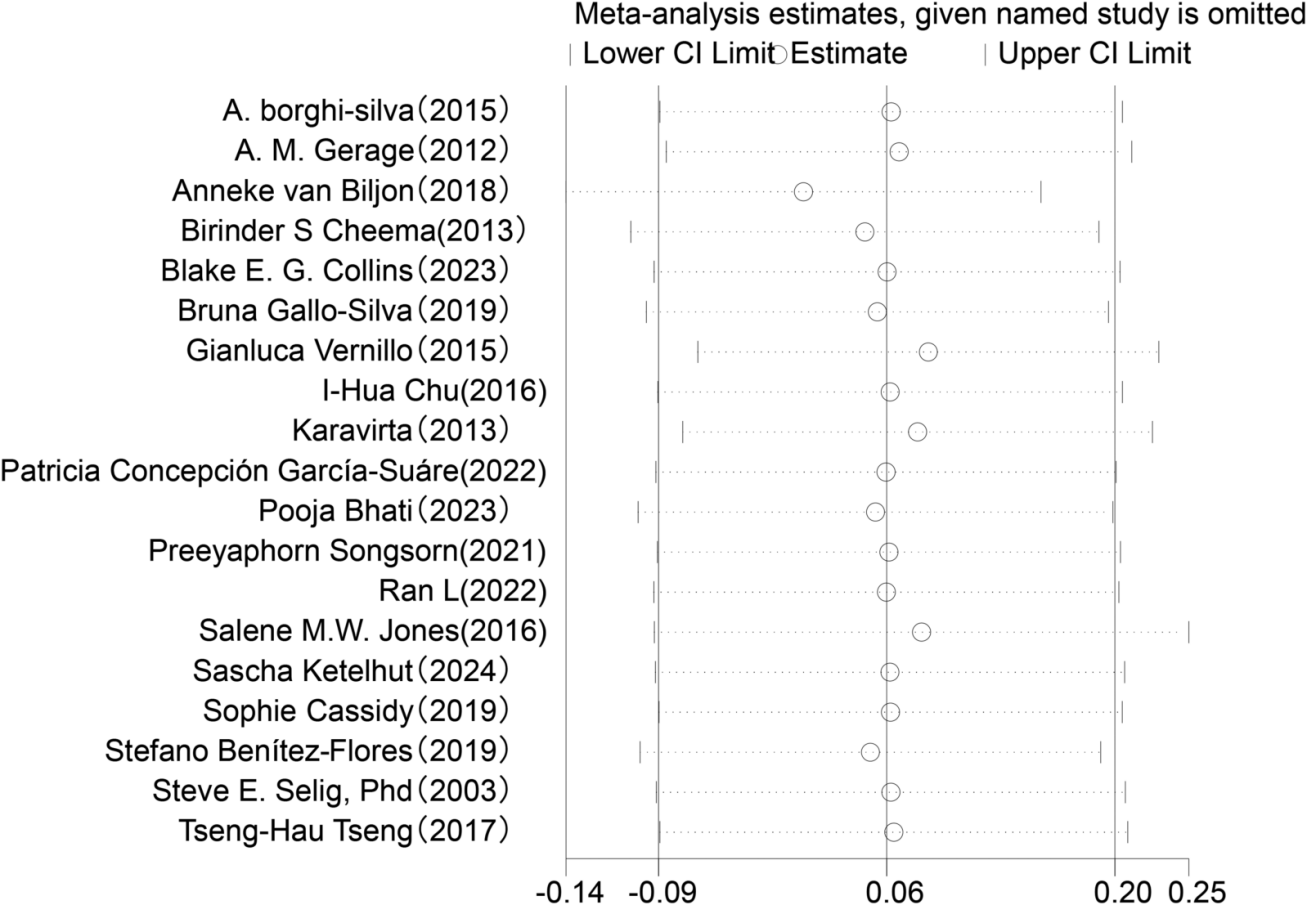
Sensitivity analysis chart of SDNN.

**Figure 10:**
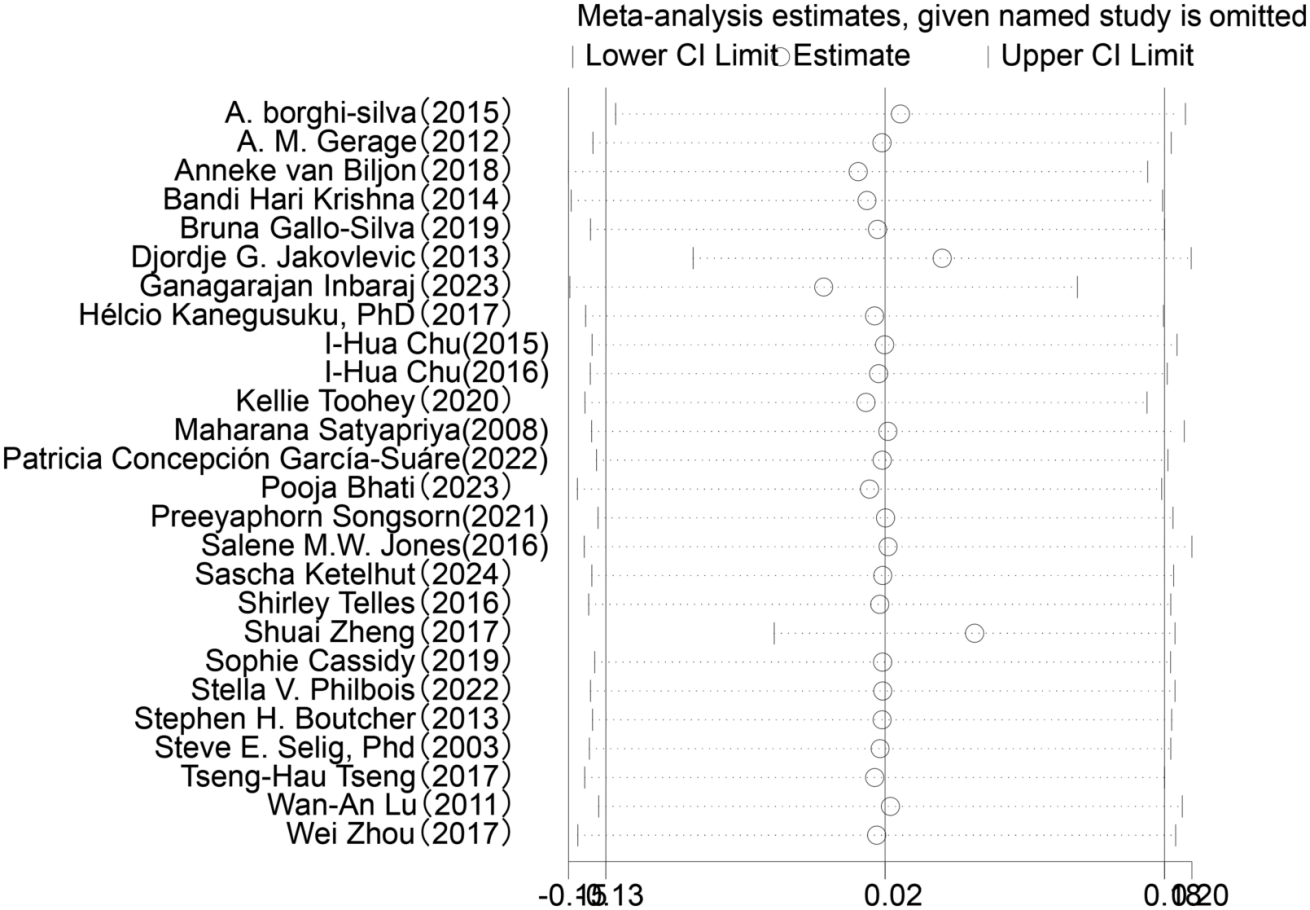
Sensitivity analysis chart of RMSSD.

**Figure 11:**
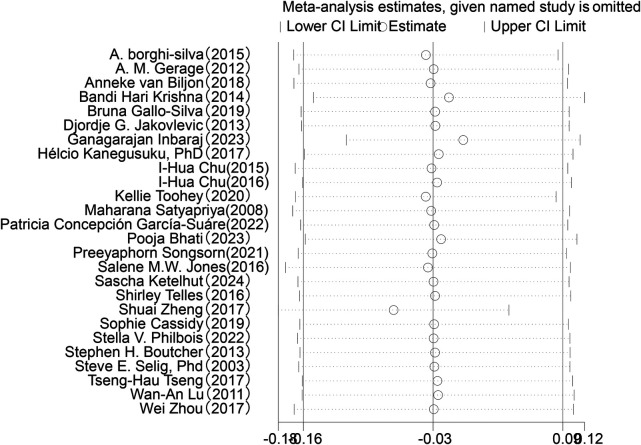
Sensitivity analysis chart of LF.

**Figure 12:**
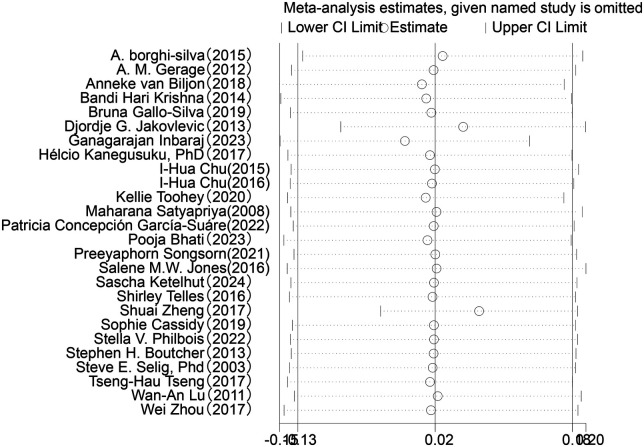
Sensitivity analysis chart of HF.

**Figure 13:**
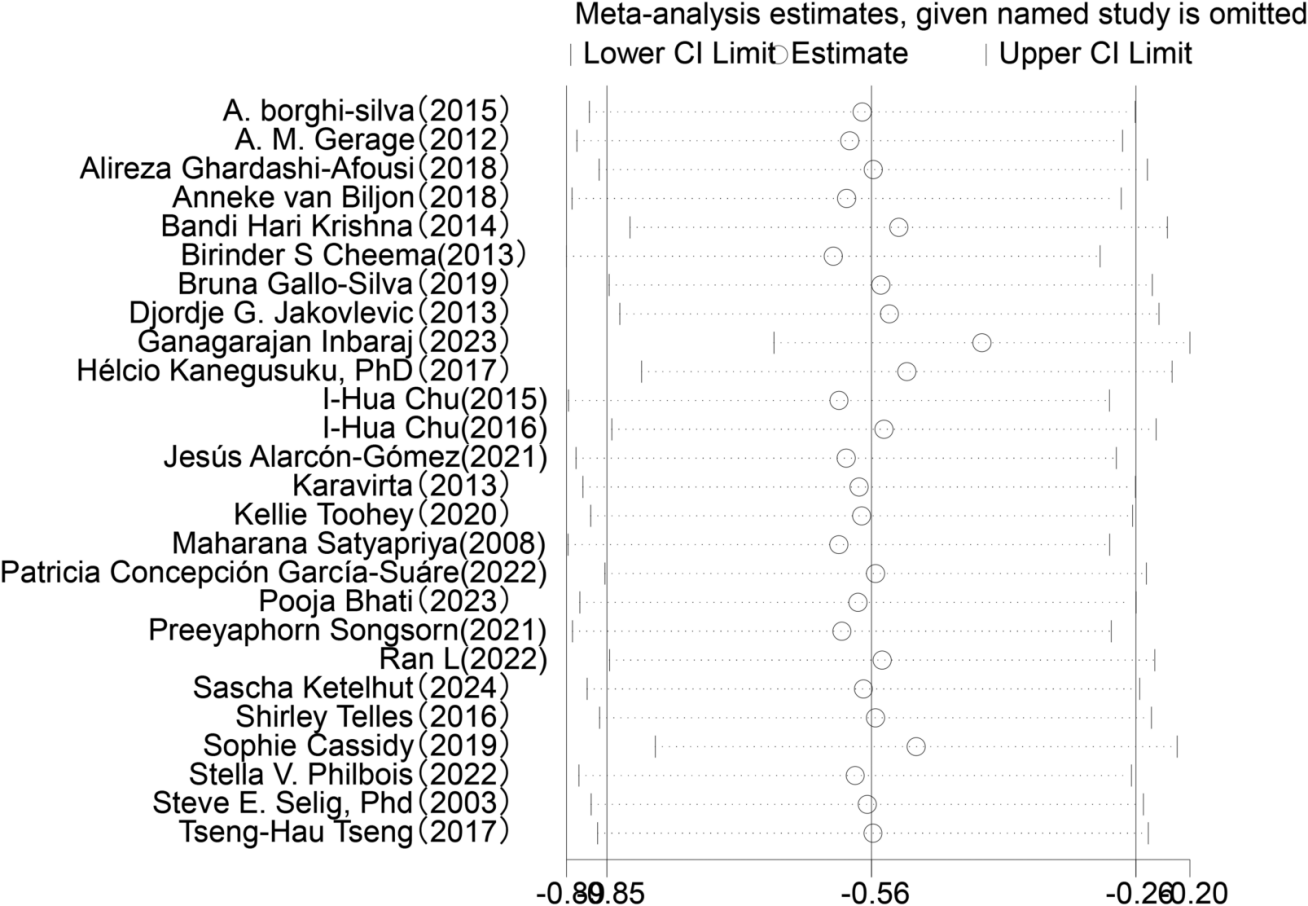
Sensitivity analysis chart of LF/HF.

Sensitivity analysis was conducted for the five HRV indices (RMSSD, SDNN, LF, HF, LF/HF) (Figures 9–13). The analysis showed that after sequentially excluding each study, the combined effect size estimates of the remaining studies all fell within the 95% confidence interval of the overall combined effect size. This finding indicates that the exclusion of any single study does not significantly alter the overall conclusions, thus confirming the robustness of the results for these five HRV indices. The sensitivity analysis charts for each index are as follows:

### 3.7 Publication bias

**Figure 14:**
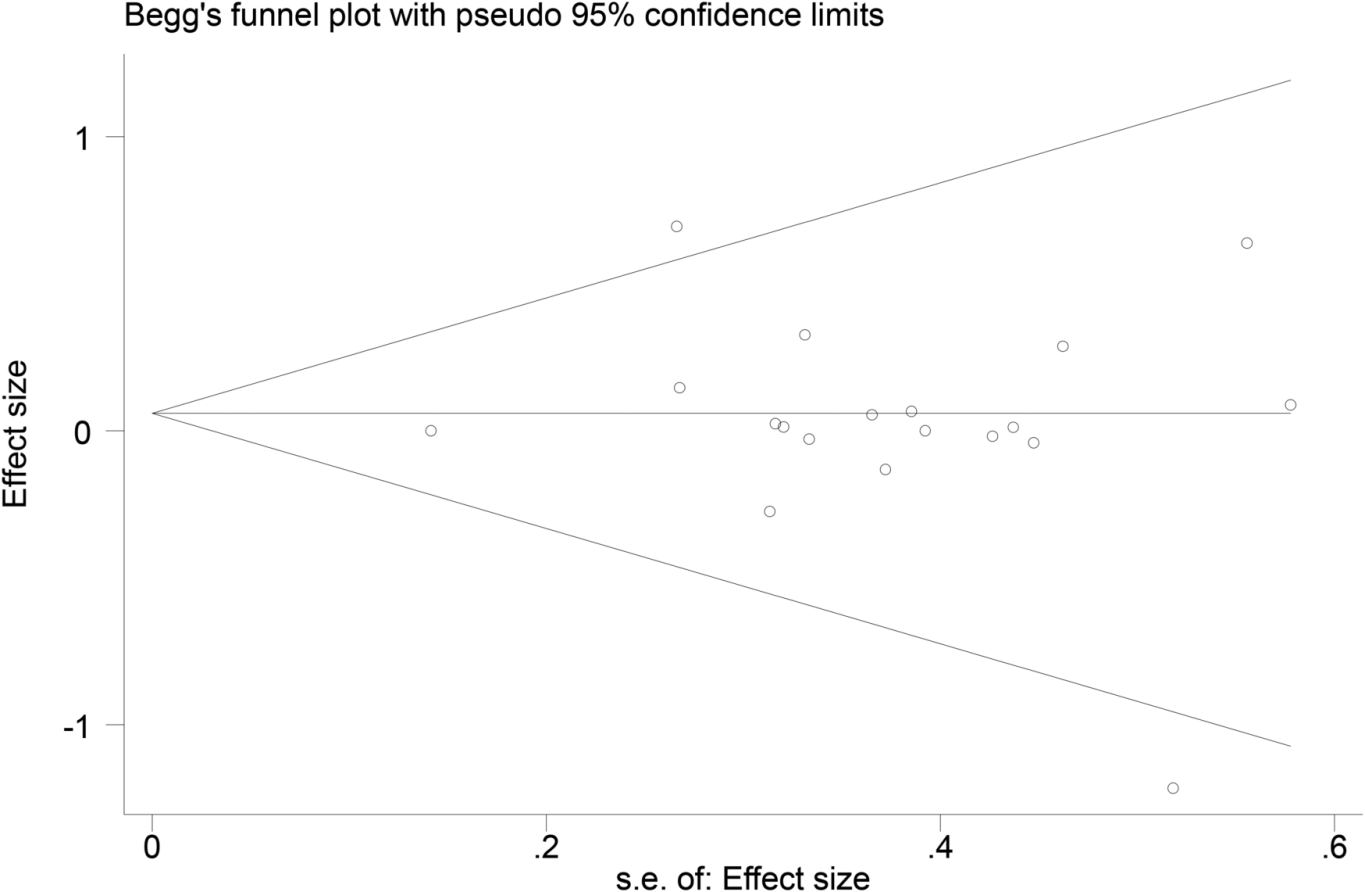
Funnel plot of SDNN.

**Figure 15:**
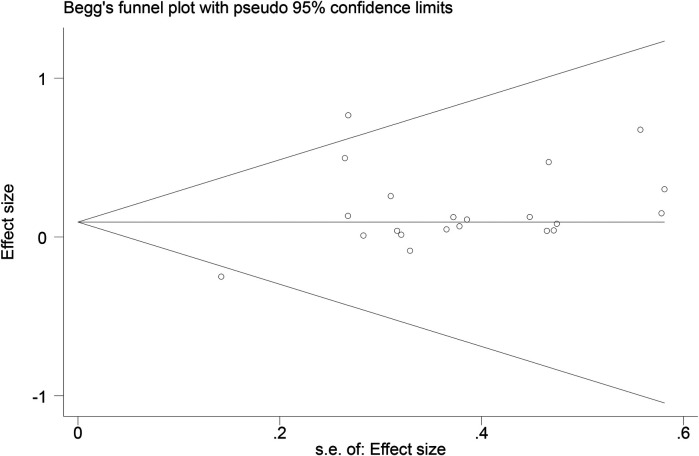
Funnel plot of RMSSD.

**Figure 16:**
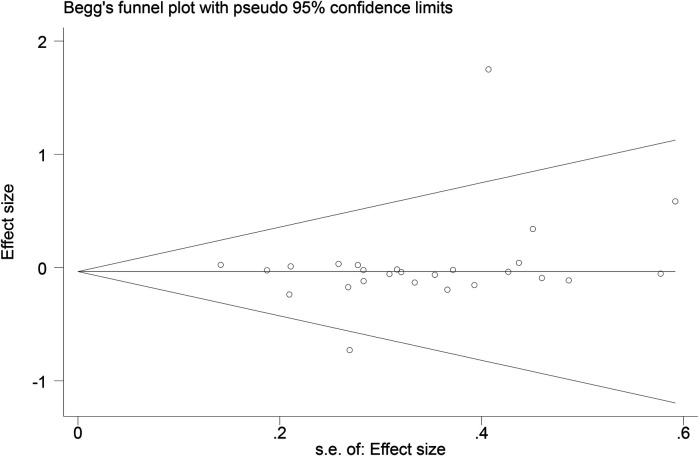
Funnel plot of LF.

**Figure 17:**
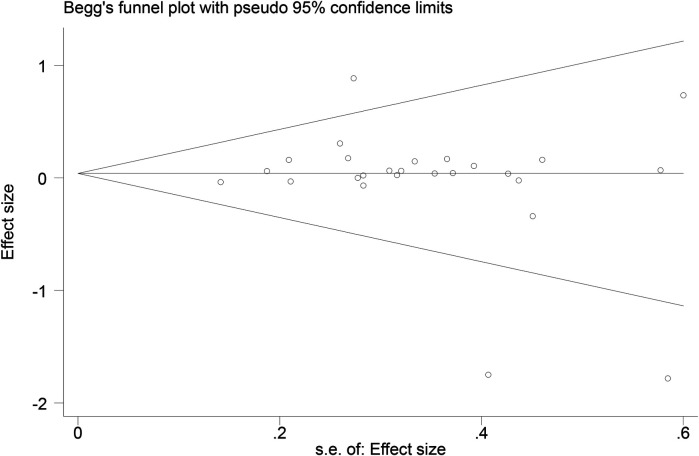
Funnel plot of HF.

**Figure 18:**
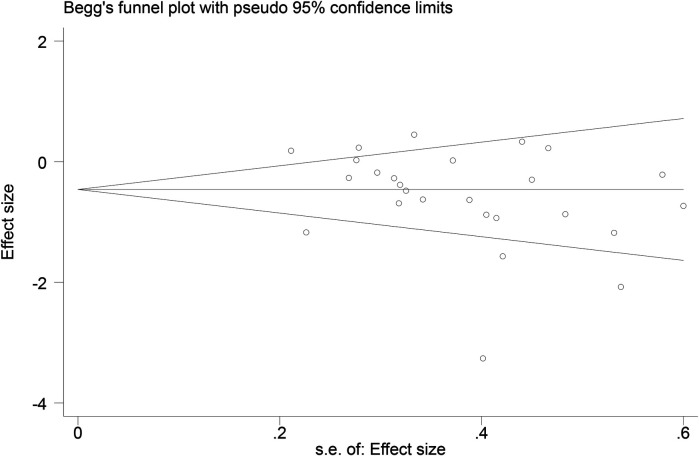
Funnel plot of LF/HF.

Funnel plots (Figures 14–18) were used to analyze the relationship between the standard error (s.e.) of SMD and SMD for different HRV outcomes, to assess the potential for publication bias. Ideally, study points should be evenly distributed around the central axis of the funnel plot, forming a symmetrical inverted funnel shape. However, the observed distribution exhibited a certain degree of asymmetry, suggesting the potential presence of publication bias. To further quantitatively evaluate bias, Begg's and Egger's tests were performed.

According to the Begg's and Egger's test results, no evidence of publication bias was found in the analyses of SDNN, LF, and HF, indicating relatively robust results. For RMSSD and LF/HF, the *P*-values were close to the significance threshold of 0.05, suggesting weak statistical evidence of publication bias. Although the asymmetry of the funnel plots does not completely rule out publication bias, its impact on the overall findings appears minimal.

## 4 Discussion

The meta-analysis indicated that long-term exercise interventions significantly reduced the LF/HF ratio, highlighting exercise's positive regulatory effects on ANS balance. A reduction in LF/HF typically reflects either inhibition of sympathetic nervous activity and/or enhancement of parasympathetic nervous activity, both of which are crucial for cardiovascular and mental health (4, 55, 56). This finding aligns with previous research. For instance, Sandercock et al.'s meta-analysis on long-term interventions involving aerobic training, resistance training, and combined training showed that exercise interventions significantly improved frequency-domain heart rate variability (HRV) metrics, particularly the LF/HF ratio (12). Similarly, Shaffer and Ginsberg (2) noted that a reduction in LF/HF not only marks decreased sympathetic activity but may also reflect enhanced vagal activation, which is essential for lowering cardiovascular disease risk and improving mental health. Furthermore, Deng et al. (57) demonstrated that exercise interventions, especially in individuals with heightened sympathetic activity or cardiovascular diseases, significantly improved HRV indices by enhancing vagal tone and inhibiting sympathetic excitability.

Despite the overall significant effects, substantial heterogeneity was observed among the studies (*I*^2^ = 77%). Therefore, this study further conducted subgroup and sensitivity analyses to explore potential sources of heterogeneity and examine the influence of different intervention types, participant characteristics, or study designs on the outcomes.

The study found that the effects of exercise interventions on LF/HF differed significantly between healthy and disease populations. Compared to the control group, the exercise intervention showed no significant effect in the healthy group, while it was markedly effective in the disease group. The between-group comparison yielded a *P*-value of 0.004, and the non-overlapping confidence intervals indicated a significant difference in intervention outcomes between the two groups. This suggests that the efficacy of exercise interventions in improving ANS function may depend on participants' baseline health status. Specifically, the pronounced improvement in the disease group may reflect more evident baseline autonomic dysfunction, such as excessive sympathetic activity or suppressed vagal tone. Exercise interventions effectively restored ANS balance in the disease group by enhancing vagal tone, suppressing sympathetic excitability, and improving metabolic health (4). For example, individuals with cardiovascular diseases, diabetes, or obesity typically exhibit lower baseline HRV and higher LF/HF ratios, making them more responsive to exercise-induced improvements (12). Additionally, exercise interventions may further optimize ANS function in the disease group by reducing chronic inflammation, improving insulin sensitivity, and enhancing cardiovascular adaptability (58). In contrast, the lack of significant effects in the healthy group may reflect their relatively balanced ANS function at baseline. Healthy individuals generally exhibit higher HRV and lower LF/HF ratios, indicating a more balanced sympathetic-parasympathetic activity (59). Thus, the limited room for improvement in ANS function in the healthy group may explain the less pronounced effects of exercise interventions. Moreover, the already healthy lifestyle and exercise habits of this population may further constrain the additional benefits of interventions.

Although significant improvements in the LF/HF ratio were observed across different age groups, the between-group comparison yielded a *P*-value of 0.94, indicating no statistically significant difference in the effects of exercise on LF/HF across age groups. This suggests that long-term exercise interventions may have a universal effectiveness in improving ANS balance across ages. However, it is noteworthy that the heterogeneity was higher in the 18–65 age group (*I*^2^ = 82%) compared to the over-65 age group (*I*^2^ = 53%). This difference in heterogeneity may reflect the diversity of participant characteristics and variability in intervention effects within different age groups. While exercise interventions were effective across age groups, the heterogeneity highlights the need for cautious interpretation of the results. For the 18–65 age group, future studies may need to refine subgroup analyses to explore the influence of participant characteristics (e.g., health status, exercise habits) on intervention outcomes. For the over-65 age group, despite lower heterogeneity, individual differences should still be considered, particularly when designing exercise interventions to ensure safety and efficacy in older adults (56).

In the mixed-gender subgroup, significant changes were observed (SMD = −0.51, 95% CI: −0.83 to −0.19, *P* = 0.002). However, in the female subgroup, the confidence interval included zero, indicating no significant effect of exercise interventions. This may be related to factors such as age, health status, or exercise type among female participants. Carter et al. (6) reported that women generally exhibit lower sympathetic responses to stress, which may result in smaller improvements in HRV following exercise interventions. This finding is consistent with Koenig et al. (60), who noted that women typically exhibit higher baseline vagal activity, potentially limiting further HRV improvements through exercise.

In the obesity subgroup, a significant reduction in the LF/HF ratio was observed, indicating that exercise interventions positively modulate ANS function in obese individuals. Obesity is often associated with excessive sympathetic activity and suppressed vagal tone, an autonomic imbalance linked to metabolic inflammation and increased cardiovascular risk (61). Exercise interventions may improve ANS function through multiple mechanisms, such as reducing metabolic inflammation, enhancing vagal tone, and suppressing sympathetic excitability, thereby restoring ANS balance. Koenig et al. (6) further supported this observation, noting that individuals with higher BMI typically exhibit lower baseline HRV, making them more sensitive to exercise-induced improvements in LF/HF. This underscores the potential importance of exercise interventions in obese populations, particularly for improving autonomic function and reducing cardiovascular risk.

However, moderate heterogeneity was observed in the obesity subgroup (*I*^2^ = 67%), suggesting variability among studies. This heterogeneity may reflect differences in health status, exercise types, and intervention adherence among obese participants. For example, obese individuals may have varying comorbidities (e.g., diabetes, hypertension) that could influence the effects of exercise interventions. Thus, while exercise interventions significantly improved the LF/HF ratio in the obesity subgroup, the heterogeneity calls for cautious interpretation of the results.

The effects of exercise interventions on LF/HF did not differ significantly across regions (*P* = 0.08). Nonetheless, *I*^2^ = 51.3% indicates moderate heterogeneity, which may reflect differences in cultural habits, exercise practices, healthcare systems, and participant characteristics (e.g., diet, physical activity levels, comorbidities) across regions.

Additionally, the results suggest that aerobic and resistance training may yield better intervention outcomes. As long as weekly exercise is maintained and the intervention duration is sufficient (≥8 weeks), both exercise types significantly improved the LF/HF ratio (aerobic training: SMD = −0.57, *P* = 0.02; resistance training: SMD = −0.56, *P* = 0.003). This indicates that aerobic and resistance training have unique advantages in modulating ANS balance. Aerobic training may optimize ANS function by enhancing cardiorespiratory fitness, improving blood circulation, and promoting vagal activity (62), while resistance training may achieve similar effects by increasing muscle mass, improving metabolic health, and reducing sympathetic excitability (12). Notably, although both exercise types showed significant effects, no significant difference was found between them (*P* = 0.84). This suggests that as long as exercise interventions meet certain frequency and duration thresholds, both aerobic and resistance training can effectively improve ANS function. This finding provides flexibility for personalized exercise intervention designs, allowing for the selection of exercise types based on participant preferences, health status, and physical capabilities.

The type of control group significantly influenced changes in LF/HF. The meta-analysis results showed that for intervention groups with a “no exercise intervention” control, the LF/HF ratio decreased significantly (SMD = −0.61, *P* < 0.001), whereas no significant change was observed for intervention groups with a “low-intensity activity” control (SMD = 0.01, *P* = 0.97). This result suggests that the improvement in LF/HF ratio when comparing exercise intervention groups to control groups may differ depending on the type of control group.Specifically, when the control group received “no exercise intervention,” the intervention group's LF/HF ratio decreased significantly, indicating a clear effect of exercise interventions on ANS balance. This significant difference may reflect the baseline state of the “no exercise intervention” control group, which was unaffected by any external intervention, thereby more clearly highlighting the positive effects of exercise interventions.When the control group engaged in “low-intensity activity,” the difference in LF/HF changes between the intervention and control groups was not statistically significant. This may be because low-intensity activity itself exerts positive effects on ANS function, thereby narrowing the gap between the two groups. This finding suggests that low-intensity exercise may, to some extent, produce effects similar to higher-intensity exercise on the LF/HF ratio. Moreover, low-intensity exercise typically has higher adherence and acceptability, making it a feasible ANS modulation strategy, particularly for older adults or individuals with poor health.

### 4.1 Study strengths and limitations

This study exhibits several strengths. First, it utilized a comprehensive and systematic approach, integrating data from multiple studies to thoroughly explore the effects of long-term exercise interventions on HRV, particularly the LF/HF ratio. Such an approach not only provides an assessment of the overall effects but also underscores the clinical significance of exercise interventions in promoting ANS balance and enhancing cardiovascular health. Second, the study analyzed not only the overall effects of exercise interventions but also systematically examined various potential moderating factors, including health status, gender, BMI, geographic location, and intervention characteristics (e.g., exercise type, duration, frequency). This multidimensional analysis offers valuable theoretical insights for designing personalized exercise intervention programs. Additionally, by conducting an in-depth analysis of intervention characteristics, the study clarified the differential effects of various intervention methods (e.g., aerobic exercise, resistance training) and intervention durations on LF/HF, thereby emphasizing the importance of careful intervention design.

However, this study has several limitations, and the findings should be interpreted with caution. First, significant heterogeneity was observed among the included studies (e.g., high *I*^2^ values), which may stem from differences in participant characteristics (e.g., health status) and intervention protocols (e.g., exercise type, intensity, frequency). This heterogeneity limits the generalizability of the results. Therefore, caution is warranted when interpreting the findings, particularly when applying them to different populations or intervention contexts. Second, although multiple methods were employed to reduce bias (e.g., sensitivity analysis, subgroup analysis), potential publication bias cannot be entirely ruled out, especially given the possible underreporting of negative results. Additionally, it is important to acknowledge that HRV, particularly the LF/HF ratio, serves only as an indirect indicator of ANS activity, reflecting correlations rather than causality, and may be influenced by other confounding factors. Finally, in the subgroup analyses, the limited number of studies in certain subgroups may have resulted in insufficient statistical power, thereby reducing the reliability of the findings. Future research should include more high-quality randomized controlled trials (RCTs) and mechanistic studies to validate these findings and further elucidate the specific mechanisms through which exercise interventions influence the ANS. Moreover, it is recommended that future studies standardize intervention protocols and measurement methods during the design and implementation phases to reduce heterogeneity and enhance the comparability of results.

## 5 Conclusion

The present study indicates that long-term exercise interventions significantly reduce the LF/HF ratio, with particularly pronounced effects observed in populations with diseases and in interventions lasting ≥8 weeks. Aerobic exercise and resistance training demonstrate strong potential for improvement.

However, these findings should be interpreted with caution. On the one hand, there is substantial heterogeneity among the included studies; on the other hand, data on certain intervention characteristics remain limited. Moreover, since the LF/HF ratio is only an indirect indicator of ANS activity, the observed correlations cannot directly establish causality. Therefore, future research should focus on conducting more high-quality studies to confirm these conclusions.

## Data Availability

The original contributions presented in the study are included in the article, further inquiries can be directed to the corresponding author.
